# The Care of the Children

**Published:** 1875-07

**Authors:** 


### The Care of the Children.

Now is the time of school vacations, when the children are let loose from the confines of school and school discipline, and allowed to enjoy an unrestricted amount of fresh air and out-door sport. The ruddy cheeks, bright eyes and rounded forms which will result will amply repay many a careful mamma for the additional care that will devolve upon her, "now that the children are out of school." The number of precious lives that are yearly sacrificed by the unwholesome confinement of schools is something frightful; and the anxious physician who is unable to persuade the parents, proud of their children's accomplishments, to take them away from school and study, will welcome the vacation as a needed adjuvant in relieving the flushed cheek and aching head—results of over study and *school air*. In a recent investigation of the Louisville, Ky., Board of Health, they discovered that the air space allowed for each scholar, in the best constructed school in the city, was less than one-seventh that required by the United States authorities for each soldier while in barracks. The greater discrepancy will be seen between these two instances when it is remembered that this amount is required by the government for brain and body-idle soldiers.

We recently asked a little girl who complained almost constantly of headache, the cause. Her reply showed a very intelligent appreciation of the situation; she said: "I have to stay in a hot school-room, and feel all the time as if I could not breathe. But, we asked, can you not get fresh air from the window? "Yes," she replied, "but then it blows from the top of the window right over our heads, and we all take cold." This school-room, the primary of one of our best schools, is crowded with children from five to ten years of age, heated by a large stove in winter, with no other means of ventilation than the windows; and in summer made dark and damp by a thick surrounding of shade trees and houses.

But the unsanitary management of our school-rooms is not the only baneful influence surrounding the health of our children. Bad air, unventilated and small sleeping apartments, poorly cooked and improper food, and injudicious but *fashionable* dress, all exercise a deleterious and frequently a lasting influence.

The wonder is not why so many children in our cities die, but how so many live at all, surrounded as they are.

The benefits of good country air and food cannot be obtained by all, but many of the evils of city life can in a measure be mitigated, a large number of them entirely done away with, and it is the duty of the physician to point out to parents and teachers the great injury which is being accomplished to the health of their young charges. The Germans apply the term *kinder-garten* to schools intended especially for young children. How different from the method pursued in a well-conducted garden is that undertaken in the care of children in our schools! Fresh air and sunlight are rigorously excluded, and the young plants are allowed to grow up pale and blighted, or die in the vain attempt to exist.

It is not alone the hygienic arrangement of the school-rooms which is to be criticised. The plan of crowding scholars to their utmost, which is pursued in many institutions, is eminently harmful. Courses of study are undertaken and completed in one-half the time which should be given to them, and in order to accomplish this result, the pupils are compelled to occupy the hours which should be given to rest and recreation, in study. The ambition to stand high on the roll of honor, or to gain the medal of merit, spurs the scholar on; but when the prize is won, can the cost in many instances be counted? The years of school life are the ones in which the body and mind are most susceptibe to injurious treatment, and parents and teachers cannot be too careful not to work a lasting injury in their ambition to make bright scholars. It is not an unfrequent thing to see young ladies taking books home to study which must occupy all their spare time. Tired out with the confinement of school, and, in many instances, the injurious practice of being compelled to pass up and down long flights of stairs, they should not be allowed tasks which they cannot finish in school hours. Dr. Clarke has shown the great injury which is wrought to the health of young ladies by overwork in certain periods of girlhood, and we earnestly wish that our educators could be made to see the injury they are working by this system of overwork and over stimulation by prizes and medals. B.

